# Approximate size and preliminary composition of the low-mass zinc pool in the cytosol of *Saccharomyces cerevisiae*

**DOI:** 10.1016/j.jbc.2026.111394

**Published:** 2026-03-20

**Authors:** Alexia C. Kreinbrink, Paul A. Lindahl

**Affiliations:** 1 Department of Biochemistry and Biophysics, Texas A&M University, College Station, Texas, USA; 2 Department of Chemistry, Texas A&M University, College Station, Texas, USA

**Keywords:** cot1, fluoZin-3, mössbauer spectroscopy, ICP-MS, sulfur metabolism, zrc1, Zn coordination complexes

## Abstract

The composition and size of the low-mass zinc pool in yeast were estimated by chromatography with inline inductively coupled mass spectrometer detection of Zn. Cytosol was isolated from cells grown with increasing Zn(acetate)_2_ and analyzed by LC-ICP-MS. Some samples were passed through a 3 kDa filter, and resulting flow-through solutions exclusively contained low-mass Zn complexes. Cytosol and flow-through solutions contained 200 to 270 μM Zn and 15 to 26 μM Zn, respectively. The latter range indicated a low-mass pool size higher than previous estimates. Cytosol chromatograms were dominated by Zn-bound protein peaks but also contained Zn complexes. Some peaks were assigned by comigration with known complexes. Accordingly, Zn-citrate likely dominated, Zn-cysteine appeared to be uniformly present; Zn-glutathione did not likely contribute. Corresponding S concentrations were higher than Zn, and most were nonproteinaceous. Yeast regulated Zn homeostasis more effectively than *Escherichia coli*, as the size of the pool increased marginally with added media Zn. Cytosol spiked with increasing concentrations of ^67^Zn(acetate)_2_ revealed that aqueous Zn binds proteins and low-mass ligands with approximately equal propensity. Cytosol bound > 15 μM of added aqueous Zn, some to available citrate. Neither Zn-bound proteins nor complexes were affected when cytosol was spiked with *FluoZin-3*; this chelator coordinated Zn from a complex that was marginally stronger than aqueous Zn. Chromatograms from cells lacking vacuolar Zn importers were similar to background WT cytosol while high Zn levels caused growth defects and changes in the pool. Mössbauer spectra of ^57^Fe-enriched cells grown with increasing Zn indicated loss of Fe/S clusters and possible formation of nanoparticles.

Zinc is essential for all living systems (1, 2, 3, 4, 5). The divalent cation (to be called Zn) is redox-inactive and often serves as a Lewis Acid at the active site of enzymes. In other capacities, it maintains protein structures such as zinc-fingers, and is involved in cell signaling, DNA replication and repair, and cell division (6). At high concentrations, Zn is toxic. Zn dysregulation is associated with numerous diseases including cancer, Alzheimer's, and diabetes (7, 8, 9). The model eukaryotic cell *Saccharomyces cerevisiae* houses hundreds of Zn proteins, corresponding to ∼ 10% of the cell's proteome (5, 10, 11, 12). Yeast cells contain > 1.5 × 10^7^ Zn atoms (13), affording concentrations > 600 μM Zn in cell volumes of about 42 × 10^−15^ L (14).

The *Labile Zn pool* in the cytosol is thought to receive Zn ions imported from the growth media and to distribute them to various species throughout the cell. Thus, these pools are considered to be the *Grand Central Station* of Zn trafficking and homeostasis. Despite their importance and decades of investigations, their chemical composition and size remain uncertain. One reason for this is the reliance on cell-permeable fluorescent probes. These probes are typically incubated with whole cells, and their fluorescent properties change upon binding Zn. These changes can be sensitively monitored and quantified. The problem is that this approach must define labile Zn pools *operationally* as whatever Zn-bound species in the cell coordinates the probe. A variety of probes have been used (15, 16, 17, 18, 19) such that the size and composition of labile pools, thus defined, differ according to the strength of the probe and reaction conditions employed. *In vitro* tests of the reactivity and specificity of such probes suggest that labile Zn pools are composed of loosely-coordinating non-proteinaceous Zn complexes with collective sizes in the femtomolar (20) to hundreds-of-picomolar (21) range.

We have recently challenged these ultra-low concentrations, due to results of a chromatography-based study in which cytosolic extracts of *Escherichia coli* were passed through either a 0.2 μm or 3 kDa cutoff filters and then separated on a LC system with real-time inline inductively coupled mass spectrometer (ICP-MS) detection (22). The former cytosol samples, called *CYT*, contain both proteins and low-molecular-mass Zn complexes; whereas the latter flow-through solutions (*FTS*s) exclusively contain low-mass Zn complexes. The concentrations of Zn in *E. coli* cytoplasm corresponded to hundreds of μM Zn, while that in FTSs was about a 10th of that – orders-of-magnitude higher than previous estimates of labile Zn pools obtained using fluorescent probes.

Chromatograms of *E. coli* CYT and FTS solutions exhibit a half-dozen Zn peaks representing a similar number of low-mass Zn species. In cytosol from cells grown on high concentrations of nutrient Zn, ESI-MS confirmed that the dominant low-mass Zn peak in LC traces (from cells grown under high Zn conditions) arose from Zn bound to glutathione (GSH). Other ligands associated with these complexes could not be identified, but cysteine seems likely (23). Zn citrate did not coelute with any of the observed species in *E. coli*. Surprisingly, > 90% of the Zn removed by the commonly used N,N,N′,N′tetrakis(2-pyridinylmethyl)-1,2-ethanediamine (TPEN) chelator probe originated from Zn-bound *proteins*. Thus, the labile Zn pool in *E. coli*, defined operationally using TPEN, mainly consists of Zn bound *proteins* rather than coordination complexes. Here we wanted to compare the low-mass Zn pool in *S. cerevisiae* with that in *E. coli*.

In an earlier investigation, Nguyen *et al.* (24) isolated CYT samples from EDTA-washed *S. cerevisiae* cells that had been grown in minimal media, and reported a Zn concentration of ∼ 500 μM for those samples. Corresponding FTSs were injected onto a size-exclusion column followed by inline ICP-MS detection. A few broad low-mass Zn peaks were observed but not identified. Their intensities were unaffected when cells were grown in medium supplemented with 100 μM Zn implying rather effective homeostatic regulation. In contrast, a Zn peak at ∼ 8500 Da, hypothesized to arise from metallothionein Crs5, increased in intensity. In the intervening years, our methods have improved, especially our awareness regarding the adsorption of metals on our chromatography columns, justifying a reinvestigation of the low-mass Zn (and sulfur) pools under different media conditions, mutant strains, and metabolic states.

A second major reason why the chemical composition of these pools remains uncertain is their inherent *lability*. This property is required for Zn to be transferred from the pools to various apo-protein clients. That same lability also promotes mismetallation and generates reactive oxygen species (25). This explains why these pools should be tightly regulated. However, we recently found that the low-mass Zn pool in *E. coli* was *not* so tightly regulated, and so here we wanted to evaluate the effectiveness of Zn homeostasis in yeast more quantitatively.

Tighter Zn homeostatic regulation would be consistent with the more extensive mechanism involved (5, 26, 27), as summarized in Figure 1. In yeast, transcription factor Zap1 is thought to sense the labile Zn pool (13). Under Zn-deficient conditions, Zap1 binds the promotor regions of over 80 genes responsible for zinc import and storage (1, 28). This includes the high-affinity importer Zrt1, low-affinity importer Zrt2 (29), and nonspecific importers Fet4 and Pho84. Besides Zn, Fet4 imports Fe, and Cu (30), while Pho84 transports phosphate and metal-phosphate complexes including Zn-phosphate (31, 32). Thus, Zn metabolism is intertwined with those of Fe, Cu, and P.

**Figure 1:**
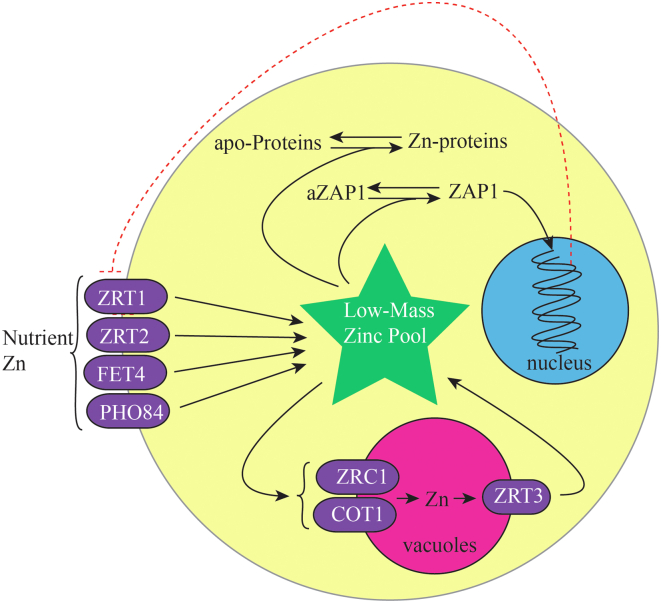
**Summary of the homeostatic regulation mechanism involving the low-mass Zn pool in *S. cerevisiae***.

Rather than exporting cellular Zn under high-Zn conditions, yeast sequesters excess Zn in vacuoles and in Crs5 (33). Under Zn-excess conditions, Zap1 is inactivated which decreases expression of importers Zrt1 and Zrt2. Vacuoles store Zn due to import *via* Zrc1 and Cot1 transporters on the membrane of these acidic organelles (34). Overexpression of Zrc1 or Cot1 increases zinc tolerance in cells (35). Under Zn-deficient conditions, Zrt3, another transporter on the vacuolar membrane, mobilizes vacuolar Zn and returns the metal to the labile Zn pool in the cytosol (13, 26, 35). Mitochondrial respiration, autophagy, and chromatin metabolism also function in Zn homeostasis (36, 37).

Here we describe the Zn (and S) content of cytosol isolated from *S. cerevisiae* cells grown under fermenting conditions in minimal media supplemented with a range of zinc acetate concentrations. Samples were injected onto a low-mass-resolving chromatography column associated with our LC-ICP-MS system. We also evaluated whether the less powerful but commonly used Zn probe *FluoZin-3* exhibits similar chelation properties as TPEN. We also spiked cytosol with aqueous Zn to examine the binding capacity of these solutions and investigate cytosol from a mutant strain of yeast in which import into vacuoles was blocked. Zn is thiophilic, such that Zn complexes with sulfur donor ligands are likely components of the low-mass Zn pool. Thus, we preliminarily characterized the low-mass sulfur species in CYT and FTS. Finally, we used Mössbauer spectroscopy to probe the effects of Zn on iron-sulfur clusters in these cells.

## Results

### Zinc and sulfur concentrations in cells, cytosol, and FTS

To characterize the low-molecular-mass (LMM) Zn pool in yeast and evaluate Zn homeostasis, *S. cerevisiae* cells were grown on minimal fermenting media supplemented with 0, 10 or 100 μM Zn(acetate)_2_ (final concentrations). Cytosol was isolated and passed through a 0.2 μm membrane filter resulting in filtrates *CYT0*, *CYT10*, and *CYT100*, respectively. These solutions were expected to contain both proteins and zinc coordination complexes. Other portions of the same isolated cytosol samples were passed through a 3 kDa membrane filter, generating flow-through solutions *FTS0*, *FTS10*, and *FTS100*. These solutions should exclusively contain LMM non-proteinaceous Zn coordination complexes.

Zn and sulfur concentrations in whole cells, CYT, and FTS solutions were determined (Table 1). Zn concentrations were highest in whole cells, next highest in cytosol, and lowest in FTSs. Those determinations indicate ∼ 200 μM Zn-bound proteins and tens-of-micromolar Zn coordination complexes in cytosol. Zn concentrations were ∼ 40% higher in CYT100 than in CYT0, and ∼ 70% higher in FTS100 than in FTS0. The Zn concentration in unsupplemented media was 1.2 μM. The modest 2-fold increase in response to what was roughly a 100-fold increase in media Zn concentration illustrates the cell's remarkable effectiveness in regulating intracellular Zn levels, including both proteins and low-mass species.

**Table 1:** Concentrations of zinc and sulfur in fermenting whole cells, isolated cytosols, and flow-through solutions

Cell fraction → Media suppl. ↓	Whole cell		Isolated cytosol		Flow-through solution	
	Zinc (μM)	Sulfur (mM)	Zinc (μM)	Sulfur (mM)	Zinc (μM)	Sulfur (mM)
1 mM EDTA	40 ± 10	32 ± 2	6 ± 0.5	14 ± 1	2 ± 0.6	20 ± 3
0 μM Zn	400 ± 50	50 ± 10	200 ± 5	32 ± 1	15 ± 7	35 ± 3
10 μM Zn	740 ± 70	70 ± 20	230 ± 20	37 ± 4	23 ± 9	46 ± 5
100 μM Zn	920 ± 80	50 ± 5	270 ± 50	37 ± 4	26 ± 8	40 ± 5
Zn saturated	-	-	200 ± 30	12 ± 1	85 ± 15	14 ± 2

Values are the average of three independent preparations. Concentrations were back-calculated to those in the cytosol of whole cells (48).

The ratios of the CYT/FTS Zn concentrations in Table 1 implies that ∼ 90% of the Zn in cytosol should be bound to proteins and ∼ 10% to nonproteinaceous coordination complexes. The substantial differences between whole-cell and cytosolic Zn concentrations may have been due to Zn in non-cytosol compartments such as vacuoles, mitochondria, or nuclei, and/or to Zn bound nonspecifically to the cell exterior. Cells were washed with HPW rather than with EDTA as is commonly done. Doing so avoided Zn-EDTA complexes from forming and contributing to the low-mass Zn pool, but it may have allowed some Zn to remain bound to the cell exterior.

Sulfur concentrations in cellular, cytosolic, and FTS samples were 2 to 3 orders-of-magnitude higher than those of Zn (Table 1), and they were nearly invariant across the series whole-cells → cytosol → FTS. This implied that nearly all sulfur-containing species in these cells had masses ≤ 3 kDa – *i.e.* they were nonproteinaceous and LMM. Sulfur concentrations were also nearly invariant across the series 0 → 10 → 100 μM media Zn. These near-invariances allowed sulfur intensities for a given chromatogram to serve as a foundational anchor for the more variable Zn intensities.

### LC-ICP-MS of CYT and FTS samples

**Figure 2:**
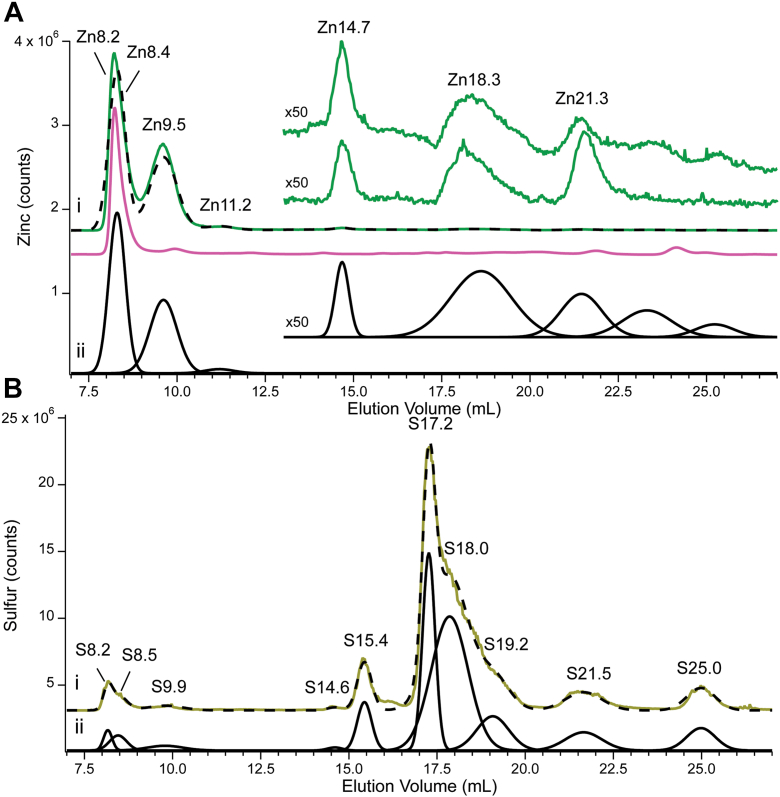
**Zinc, A280, and sulfur-detected chromatograms of CYT0 and FTS0.***Panel A,* Zn (*green*), A280 (*pink*), individual simulations (*black*) and cumulative simulations (*black dashed*) (*i*) CYT0 with a zoom-in of CYT0 in the non-protein region (upper trace) and FTS0 (lower trace), (*ii*) CYT0 individual peak fits; *Panel* B: S (*yellow*). (*i*), CYT0, (*ii*), CYT0 individual peak fits. Each chromatogram is the average of traces from three independent batches. Multiplication factors: Zn 1×, zoomed and FTS0 as indicated, A280 150,00×, ^32^S 1x. The same color-coding was used for all figures.

**Figure 3:**
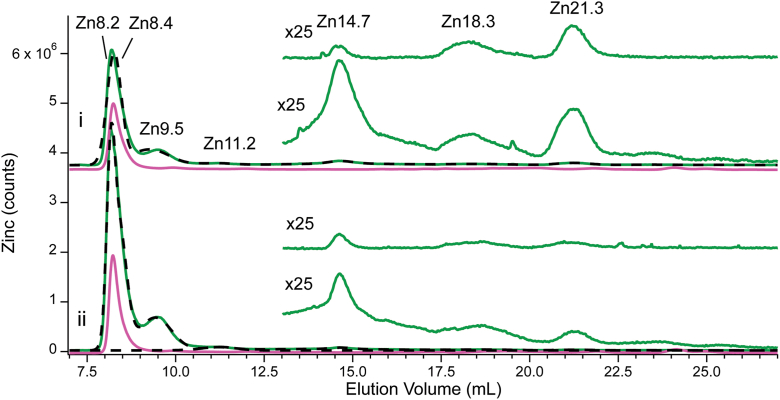
**Zn- and A280-detected chromatograms of CYT10, CYT100, FTS10, and FTS100.***Panel i*: Full *green line* (7–27 ml) is the Zn trace of CYT10. Top *shorter green line* (13–27 ml) is the Zn trace of CYT10 multiplied 25×. *Bottom short green line* is the Zn trace of FTS10 multiplied 25×. *Black dashed line* is the overall simulation, using terms and parameters in SI Data and Fits. Pink line is A280 of CYT10 multiplied 15,000×. Panel *ii*: Same as in Panel *i* except using CYT100 and FTS100. Each chromatogram is the average of traces from 3 independent batches.

CYT and FTS solutions were injected onto a low-mass-resolving size-exclusion column. Eluates were monitored for absorbance at 280 nm using an inline diode array detector, and for Zn and S by inline ICP-MS. A280 was used to assess protein distributions in eluates. Resulting chromatograms were fitted as a sequence of Gaussian peaks, each defined by elution volume, intensity/counts, and linewidths (full width at half maximum). CYT0 and FTS0 chromatograms are shown in Figure 2 (Zn in top panel and S in bottom panel) whereas CYT10, CY100, FTS10 and FTS100 are shown in Figure 3. Peak positions varied slightly from one batch or one column to the next; we generally assigned peaks that had ± 0.1 ml of the same elution volume to the same Zn- or S- containing species (see exact ranges in Data and Fits). We also considered the similarity of linewidths in deciding whether two peaks from different traces arose from the same species. Peaks were labeled according to the element detected and average elution volume; for example Zn14.7 refers to a Zn peak at 14.7 ml.

One unavoidable problem was that a significant fraction of the Zn in CYT and FTS samples adsorbed onto the column. To quantify this, aliquots of the same samples were injected onto a “ghost” column in which peek tubing replaced the column. The overall eluted Zn intensities associated with the actual chromatograms were divided by the corresponding intensities from the ghost column and multiplied by 100 to yield the percent of Zn that eluted. For the series CYT0 → CYT10 → CYT100, such percentages were 30%, 24%, and 33%, respectively. The fraction that did not elute is called “*ghost Zn*”. Two factors governed the percentages of eluted Zn. One factor was that the stronger (weaker) that the migrating protein or complex bound Zn, the less (or more) that it interacted with the column. For example, nearly 100% of Zn-TPEN, a strong Zn coordination complex, eluted whereas nearly 100% of aqueous Zn adsorbed onto the column. The second factor was that samples with higher Zn concentrations adsorbed less (percentagewise) than those at lower concentrations. Concentrating samples by lyophilization initially appeared to be a viable solution to this column-adsorption problem, but doing so altered the distribution of both Zn-bound and sulfur low-mass species, and so was abandoned. A related problem was that some ghost Zn eluted from the column during the subsequent injection. Such phantom appearances were minimized by injecting chelator-cocktail after every sample injection, followed by high-purity water to condition the column for the next sample injection. However, this slowed the processing of samples (each requiring ∼ 3 h) and was not fully successful in eliminating phantom peaks. The best solution was to replace the columns more frequently than one might expect or desire. This tedious and meticulous approach was employed for all chromatograms obtained in the study. Despite these problems, our results could be organized into a consistent set of Zn (and S) peaks as summarized in SI Data and Fits. Zn peak intensities, when normalized to the total intensity, yielded a coefficient of variation of ∼ 15% which we regard as excellent reproducibility, given said problems.

*Absolute* peak intensities are also given in the SI. However, the greater variability of absolute intensities (compared to relative intensities) added to the difficulty of identifying trends. For example, total absolute Zn counts of the CYT0 → CYT10 → CYT100 chromatograms increased ∼ 50% across this series, and this was consistent with the increases in determined Zn concentrations across the series (Table 1). However, with only 9 LC-ICP-MS traces included in this comparison (each displayed chromatogram is the average of three independent traces), and 23% coefficient-of-variation, we were not confident that the overall increase was significant. Moreover, no such increase was observed in the FTSs across the same series. In any event, we can confidently conclude that *yeast cells regulate Zn cellular levels effectively; overall Zn levels in the cytosol and FTS increased marginally, if at all, when cells were exposed to nearly two orders-of-magnitude increase of Zn in the growth media*.

All CYT chromatograms across the series were dominated by four peaks (Zn8.2, Zn8.4, Zn9.5 and Zn11.2) due to Zn-bound proteins (Figs. 2 and 3, and Data and Fits). These peaks collectively represented 87% of total Zn intensity. Yeast cytosol contains many (likely hundreds of) Zn proteins that overlap to generate those four peaks. The remaining 13% of Zn intensity was due to between three and nine low-mass species, eluting from 14.7 ml – 35 ml that undoubtedly arose from Zn coordination complexes. Each of those peaks represented between 0.2% and 4.5% of total Zn intensity. The corresponding FTS chromatograms (Figs. 2 and 3, lower insets) exhibited three major low-mass Zn peaks, namely Zn14.7, Zn18.3, and Zn21.3. FTS0 and FTS10 also exhibited a broad fourth peak at ∼ 32 ml. CYT traces exhibited additional broad low-intensity peaks ranging from 23 to 35 ml, but there was significant prep-to-prep variation in this group. Such broad peaks reflect weakly-bound readily exchangeable Zn complexes that interact significantly with the column. They are not aqueous Zn ions, which are fully adsorbed by the column, but they are only marginally different.

Sulfur-containing compounds eluted with essentially no column adsorption, and peak reproducibility was excellent. CYT chromatograms exhibited 10 peaks (S8.2, S8.5, S9.9, S14.6, S15.4, S17.2, S18.0, S19.2, S21.5, and S25.0) (Fig. 2, bottom panel, and Data and Fits). The first three peaks were due to cytosolic S-containing proteins while the others arose from low-mass sulfur compounds. A few S species comigrated with Zn complexes (Zn18.3 with S18.0; Zn21.3 and S21.5; Zn25.4 and S25.0) but comigration might be coincidental. Of clear difference, relative to Zn, was that *only 5% of total S intensity was due to S-bound proteins; ∼ 95% was due to low-mass species*. This surprising result was confirmed by the results of Table 1 which show about the same concentrations of sulfur in whole cells, CYT, and FTS samples. FTSs across the series exhibited the same set of low-mass peaks as CYT chromatograms, as well as an additional peak, S16. In all chromatograms, the central group of peaks (S17.2, S18.0, and S19.2) dominated, representing ∼ 75% of total sulfur intensity.

### Spiking FTS10 with candidate ligands

**Figure 4:**
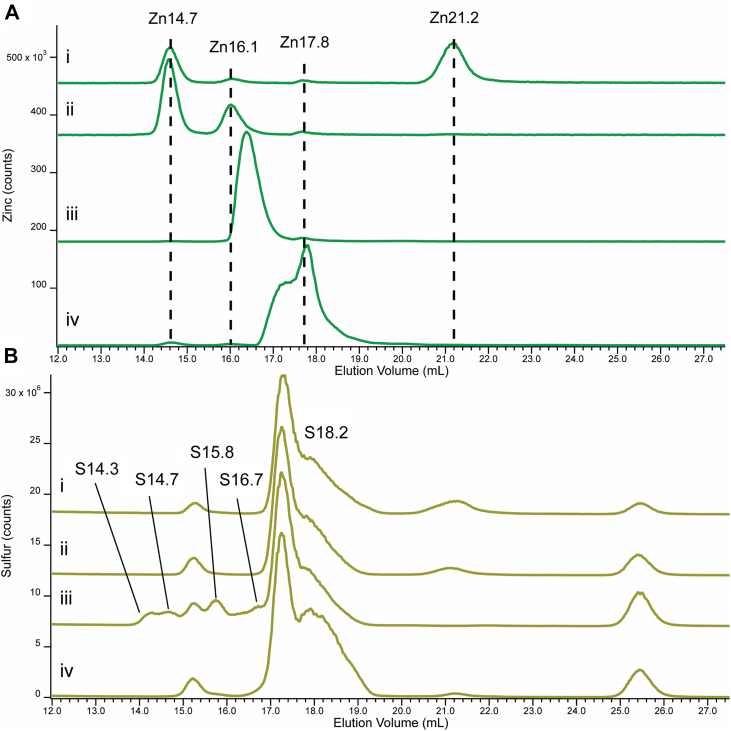
**Zinc- and sulfur-detected chromatograms of FTS10 spiked with three candidate ligands.***Panel A*, FTS10 plus 1 mM *(i)*, nothing, (*ii*) citrate, (*iii*) glutathione, and (*iv*) cysteine. *Panel B,* same as A but sulfur detection. Multiplication factors: (*i-ii*), Zn 1×, (*iii-iv*) Zn 0.1×, ^32^S 1x.

Citrate, glutathione, and cysteine (1 mM each, final concentrations) were individually added to a FTS10 sample to help identify the observed LMM peaks. Prior to spiking, the FTS10 chromatogram (Fig. 4, top panel, *i*) exhibited 4 Zn peaks (Zn14.7, Zn16.1, Zn17.8, and Zn21.2), with Zn21 dominating. After spiking with citrate (Fig. 4, top panel, *ii*), Zn14.7 and Zn16.1 increased majorly, Zn17.8 was unaffected, and Zn21.1 disappeared. As a result, Zn14.7 and Zn16.1 were assigned to different Zn-citrate complexes, and Zn21.1 was assigned to a weak easily exchangeable Zn coordination complex. Upon citrate spiking, the Zn in Zn21.1 must have transferred to citrate. There were no major changes in the corresponding sulfur chromatograms (Fig. 4, bottom panel, *i versus ii*) which was expected since citrate doesn't contain sulfur.

The FTS10 sample spiked with GSH exhibited a broad intense peak centered at 16.4 ml that represented 93% of total Zn intensity (Fig. 4, top panel, *iii*). We fitted the broad peak with two sharper peaks assigned to different Zn-GSH complexes. As was the case with citrate spiking, the Zn21.2 peak disappeared after adding GSH, consistent with our suggestion that Zn21.2 is a weak coordination complex such that its Zn was transferred to the added GSH.

The corresponding sulfur trace for the GSH-spiked sample (Fig. 4, bottom panel, *iii*) was similar to the FTS10 control, except for the appearance of four additional peaks (S14.3, S14.7, S15.8 and S16.7) and the disappearance of S21.2. The first two of these peaks likely arose from GSSG, as similar peaks were observed previously from this species (22). The next two peaks (S15.8 and S16.7) were likely due to GSH.

The absence of these peaks in control sulfur traces suggests the absence of GSH in unspiked CYT (Fig. 2, bottom panel) and FTS samples (Fig. 4, bottom panel, *i*). This is difficult to reconcile with the 1 to 10 mM GSH intracellular concentrations reported for cells (38), but consider the following. First, the overall sulfur concentration in FTS (Table 1) was unexpectedly high such that a few mM contributions would only represent ca. 10% of the total. Second, GSH degrades in *S. cerevisiae* with a half-life of 90 min (38). Processing of our samples required multiple half-lives, which would have lowered GSH concentrations to a fraction of their original values. Third and finally, GSH concentrates in vacuoles, such that cytosolic concentrations might be lower than vacuolar ones. Clearly Zn-GSH complexes are stable, and GSH can displace Zn from other complexes (*e.g.,* Zn21.2). All things considered, we suggest that the concentration of Zn-GSH in intracellular cytosol is substantially higher than reflected in our chromatograms.

The Zn trace of FTSs that were spiked with cysteine exhibited intense Zn17.2 and Zn17.8 peaks (Fig. 4, top panel, *iv*). We assign these peaks to Zn-cysteine complexes. The corresponding S trace (Fig. 4, bottom panel, *iv*) exhibited a more intense S18.2 peak which is probably uncoordinated cysteine.

### Spiking of CYT10 and FTS10 with ^67^Zn(acetate)_2_

**Figure 5:**
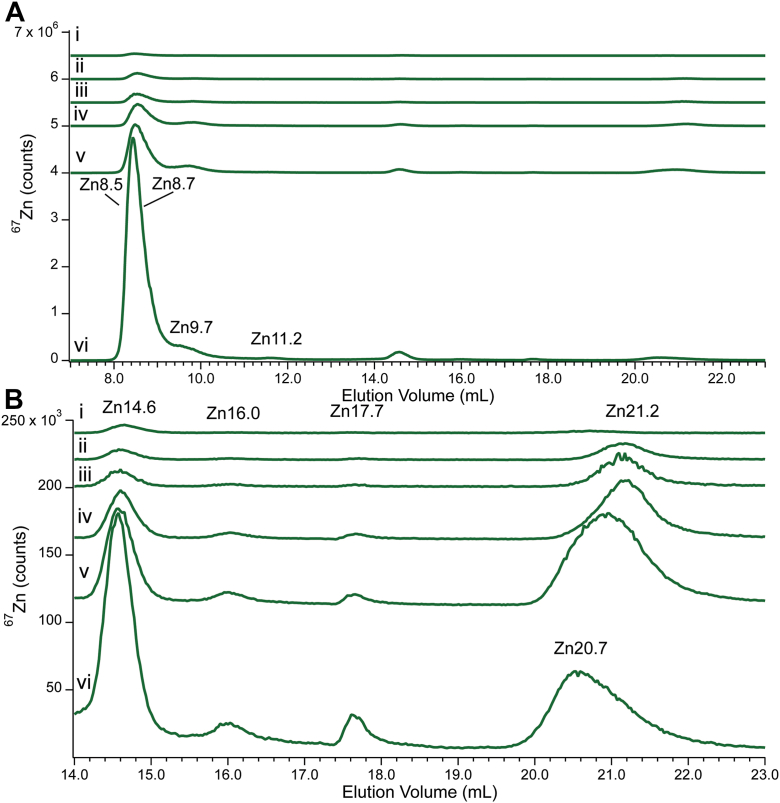
**Zinc-detected chromatograms of CYT10 spiked with ^67^Zn(acetate)_2_.** (*i*), CYT10; (*ii-vi*), CYT10 plus the following final μM concentrations of ^67^Zn: (*ii*) 2, (*iii*) 5, (*iv*) 10, (*v*) 20, or (*vi*) 50. Multiplication factors: (*i-vi*), Zn 1×.

Aliquots of CYT10 were spiked with ^67^Zn(acetate)_2_ at increasing final concentrations of 2, 5, 10, 20 and 50 μM. The original trace (Fig. 5*A*) exhibited the standard Zn-bound protein peaks (Zn8.5, Zn8.7, Zn9.7, and Zn11.2) and four more due to Zn complexes (Zn14.6, Zn16.0, Zn17.7, and Zn21.0; expanded in Fig. 5*B*). The proteins represented 84% of total intensity while the complexes represented the remaining 16%. All peak intensities increased with added ^67^Zn, roughly in proportion to the original intensity of the peak (*i.e.,* the intensity of the more intense peaks increased to a greater extent than low intensity peaks). The Zn21 peak shifted during the titration, between 21.2 ml to 20.7 ml, suggesting multiple species. Overall, 93% of the growth occurred in protein peaks and 7% in Zn complexes. Thus, we concluded that *aqueous* ^*67*^*Zn binds both Zn apo-proteins and low-mass Zn-binding ligands with approximately equal propensity*.

These results demonstrate that the *cytosol has the capacity to bind between 15 μM to > 50 μM of aqueous Zn.* The wide range arises because only ∼ 30% of the added ^67^Zn eluted from the column in the spiked sample. The lower estimate assumes that cytosol only had the capacity to bind 30% of the added 50 μM ^67^Zn whereas the upper limit assumes that all added ^67^Zn bound sites in cytosol but that only 30% of those remained bound as it migrated through the column. In any event, the cytosol has the capacity to coordinate *at least* the same concentration of aqueous Zn as is present as low-mass Zn complexes in our FTSs. This explains why the concentration of *aqueous* Zn in the cytosol is exceedingly low (*e.g.* fM or pM).

Corresponding sulfur traces exhibited excellent reproducibility, with the same set of peaks observed for all concentrations of ^67^Zn added, affording a coefficient of variation of 15%. Dominant peaks were S17 and S18, collectively representing ∼ 60% of total intensity. Similar to the sulfur traces described above, sulfur-bound proteins represented a very small percentage (in this case 4%) of total sulfur intensity, whereas low-mass sulfur species represented 96% of the total.

**Figure 6:**
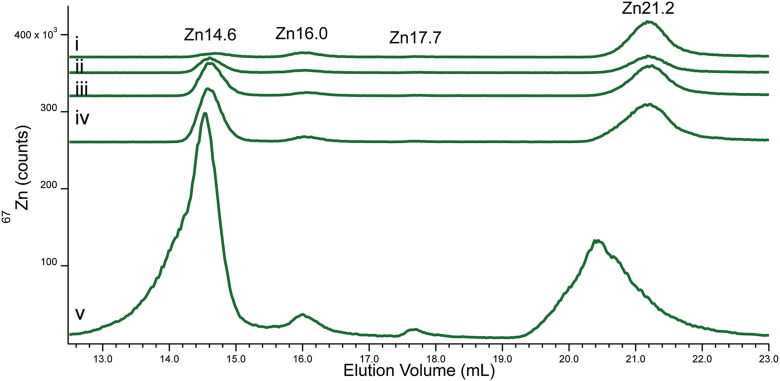
**Zinc-detected chromatograms of FTS10 spiked with ^67^Zn(acetate)_2_.** (*i*), FTS10; (*ii-vi*), FTS10 plus the following final μM concentrations of ^67^Zn: (*ii*) 2, (*iii*) 5, (*iv*) 10, (*v*) 20, or (*vi*) 50. Multiplication factors: (*i-vi*), Zn 1×.

A similar spiking experiment was conducted using FTS10, in which 0, 2, 5, 10, and 50 μM ^67^Zn(acetate)_2_ were added. Prior to addition, the chromatogram (Fig. 6, *i*) exhibited a similar set of peaks (Zn14.6, Zn16.0, Zn17.7, and Zn21.2), with Zn21.2, representing ∼ 75% of total Zn intensity. The pattern observed for the FTS10 spiking experiment was remarkably similar to that for CYT10 spiking (compare Fig. 5*B* to Fig. 6). Apart from a slight decline in total intensity when 2 μM ^67^Zn was added to FTS10, the total ^67^Zn intensity in the other FTS10 traces climbed across the series. Of this, about half of the total growth was with Zn14.7 assigned to Zn-citrate. In general, only ∼ 7% of Zn in FTS eluted from the column (93% ghost Zn) on average, so it is difficult to quantify the capacity of FTS for added aqueous Zn. However, the FTS alone, including Zn-binding low-mass ligands but not proteins, has some (μM range) capacity to bind aqueous Zn. Much of this capacity appears to be due to available citrate or other ligands eluting at that position.

Corresponding sulfur traces for the FTS10 spiking experiment were highly reproducible, affording a coefficient of variation of just 1%. The same set of peaks was present in each trace, namely S15.3, S17.3, S17.8, S18.6, S21.1, and S25.5 (not shown but see SI Data and Fitting). S17.3 and S17.8 together represented 74% of total sulfur intensity.

### Spiking CYT10 with FluoZin-3

**Figure 7:**
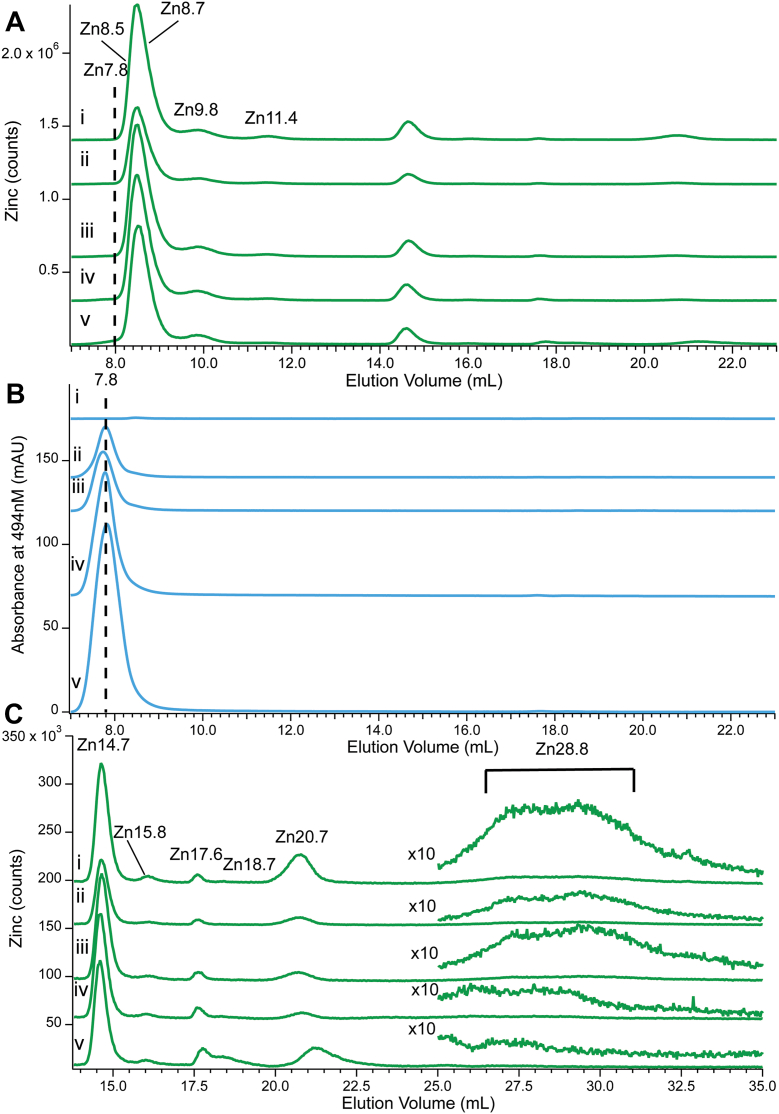
**Zinc and A494 detected chromatograms of CYT10 spiked with FluoZin-3.***Panel A,* Zn detection of CYT10 plus FluoZin-3 at the following final concentrations in μM: (i) 0, (*ii*), 13.3, (*iii*) 20, (*iv*) 50, or (*v*) 100. The *dashed vertical line* indicates the position of Zn-bound FluoZin-3. *Panel B*, same as *A* except monitored at 494 nm. Multiplication factors: Zn 1×, A494 1x. *Panel C*, same as A except focused on the low mass region, including Zn28 (intensity in that region multiplied by 10).

Prior to adding FluoZin-3, the CYT10 sample used in this experiment exhibited the standard set of Zn-protein peaks (Zn8.5, Zn8.7, Zn9.8 and Zn11.4) and low-mass peaks (Zn14.7, Zn15.8, Zn17.6, Zn18.7, and Zn20.7) (Fig. 7*C*). An additional broad low-intensity peak was observed (Zn28.8) which was sporadically present in traces from other experiments. About 80% of total Zn intensity was due to the protein peaks, 14% to the standard group of Zn complexes, and 6% to Zn28.8. The broadness and large elution volume of this last peak suggested that it arose from a weakly coordinated Zn complex that interacted to a significant degree with the column. The Zn28.8 species was not aqueous Zn (which fully adsorbs on the column) but is near to it in terms of strength of ligand-binding.

Aliquots of the same CYT10 sample were treated with 13, 20, 50 and 100 μM FluoZin-3 (final concentrations), and then injected onto the column. Overall Zn intensities did *not* display any obvious trend across this series (Fig. 7*A*); the intensity of the proteins and most coordination complexes did not change. Thus, *neither Zn ions that were bound to proteins or to most low-mass complexes were susceptible to FluoZin-3 chelation*. However, the Zn28.8 intensity decreased from 6% to 1% of total eluted Zn across the series (Fig. 7*C*).

This was somewhat counterbalanced by an increase in a *barely* observable peak labeled Zn7.8 (Fig. 7*A*, dashed line) due to Zn-bound FluoZin-3. Zn7.8 intensity increased from 0% to 1.5% of total intensity across the series. Zn7.8 was assigned to Zn-bound FluoZin-3 due to the intense A494 absorption peak at 7.8 ml (Fig. 7*B*). The A494 peak intensity was remarkably sensitive relative to that of Zn7.8 (0.77 milliabsorbance units increase per μM FluoZin-3 added). We conclude that FluoZin-3, at a concentration of 100 μM, bound only ∼ 1.5% of the total Zn in yeast cytosol, and then only to the most weakly coordinated form of Zn only marginally stronger than aqueous Zn.

### LC-ICP-MS of cytosol from zrc1cot1ΔΔ cells

**Figure 8:**
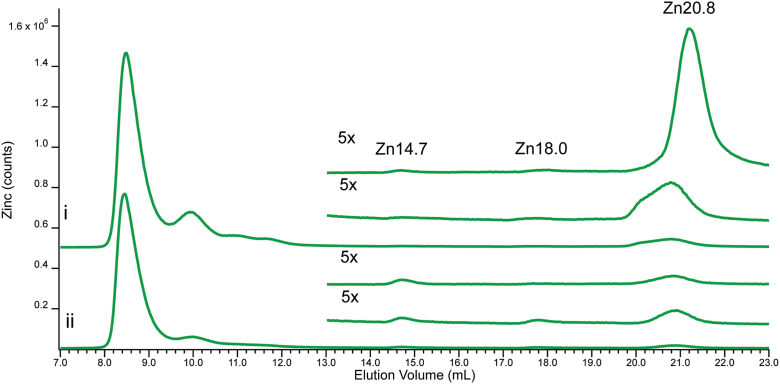
**CYT50 and FTS50 from CM100 and CM104 cells grown in media supplemented with 50 μM Zn(acetate)_2_.** Trace (*i*), CYT50 isolated from CM100 cells with CYT50 zoomed (*lower*) and FTS50 from CM100 cells (*upper*). Trace (*ii*), CYT50 isolated from CM104 cells with CYT50 zoomed (*lower*) and FTS50 from CM104 cells (*upper*). Multiplication factors: (*i-v*), Zn 1×, zoomed 5×.

We wondered whether the absence of both vacuolar Zn importers would perturb the Zn content of the cytosol of the mutant cells when grown on media supplemented with near toxic levels of Zn(acetate)_2_. To investigate, *zrc1cot1ΔΔ* cells (strain CM104) and the background strain (CM100) were grown on media containing 50 μM Zn(acetate)_2_. Mutant cells could not grow with higher Zn concentrations. CYT and FTSs from these cells (called CYT50 and FTS50) were subjected to LC-ICP-MS analysis. The resulting Zn-detected chromatograms were qualitatively similar (Fig. 8 *i* vs. *ii*), with Zn-proteins representing ∼ 93% of total Zn intensity and Zn complexes representing the remaining ∼ 7% (mainly Zn14.7, Zn18.0, and Zn20.8). Contrary to our expectations, the overall Zn intensity of the background WT strain was somewhat higher than that of the mutant, but the significance of this, if any, is uncertain. Corresponding sulfur chromatograms were similar in terms of peaks and intensities. We conclude only that yeast cells maintain a rather consistent cytosolic low-mass Zn pool, in terms of composition and size, even when Zn from that pool is blocked from entering vacuoles for storage. Probing zinc speciation in the vacuole or other compartments may shed further light on the effects seen in the cytosol.

### Testing the extremes

We wondered how the size of the low-mass Zn pool would be affected by growing cells under more extreme Zn-deficient vs. Zn-excess conditions. To do this, we initially grew WT cells under such conditions, as defined by Eide (35, 39). Zn-deficient conditions were achieved by including 1 mM EDTA and 20 mM citrate to the growth medium. For Zn-excess conditions, 6 mM Zn(acetate)_2_ was included. However, with our fermenting minimal media, the Zn-deficient cells did not grow. They did grow when 1 mM EDTA alone was included, and so we used that condition instead. Cells grew with 6 mM Zn(acetate)_2_ added to the media, but upon harvesting, the pellet included a precipitate (likely a Zn salt) that was not observed under other conditions. We consider those conditions *Zn-saturated*.

**Figure 9:**
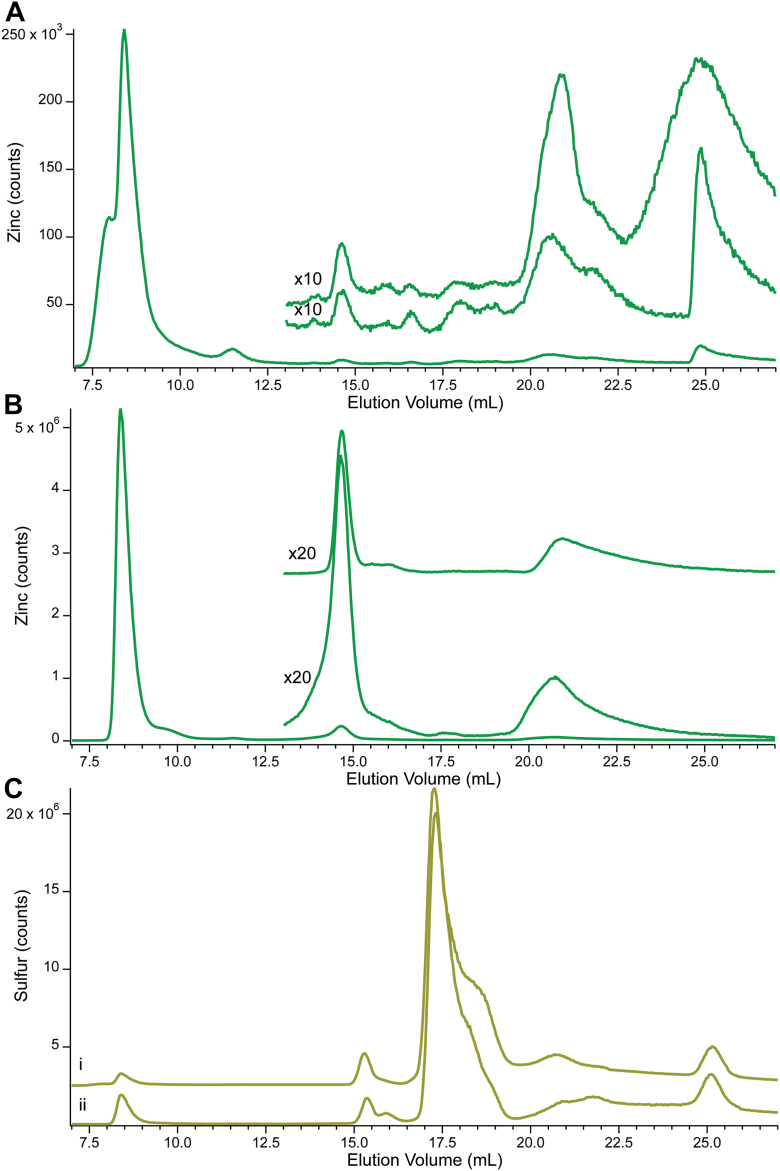
**Zn-detected chromatograms of Zn-deficient and Zn-saturated CYT and FTS samples.***Panel A*: Full *green line* (7–27 ml) is the Zn trace of Zn-deficient CYT samples. *Bottom* shorter *green line* (13–27 ml) is the Zn trace of the same but multiplied 10×. *Top* short *green line* is the Zn trace of Zn-deficient FTS multiplied 10×. *Panel B*: Same as in *Panel A* except using Zn-saturated samples. Multiplication factors for *bottom short* trace and *top short* trace are both 20x. Panel *C*: Sulfur traces from (*i*) Zn-deficient and (*ii*) Zn-saturated CYT samples. FTS, flow-through solution

Cytosol and FTSs were isolated from three independent replicates for each condition. The LC-ICP-MS traces of the Zn-saturated samples exhibited Zn protein peaks and low-mass Zn peaks similar to other samples (Fig. 9*B*). Overall intensities were similar to those obtained under milder conditions, including the proportion due to proteins vs low-mass species (88% vs 12%). Zn concentrations were qualitatively (but not quantitatively) consistent with the Zn concentrations obtained for other samples (Table 1).

The LC-ICP-MS traces of the Zn-deficient samples (Fig. 9*A*) also showed a similar overall distribution of proteins vs complexes (81% proteins; 19% complexes) but a larger number of low-mass Zn species were observed, albeit with minor intensities that might have been obscured in the more intense Zn saturated trace. Overall Zn intensities of the Zn-deficient traces were only ∼ 9% of the Zn-saturated traces. These differences were not due to instrument variations, as the overall intensities of the corresponding sulfur traces were identical within 1%. Most of the overall decline was due to a decline of Zn-protein intensities; the overall low-mass Zn pool intensities were less affected. This might suggest that the low-mass Zn pool is regulated more effectively than Zn bound proteins. This analysis was qualitatively (though not quantitatively) consistent with measured Zn concentrations of these samples in Table 1.

### Low-mass Zn pool in whole-cell extracts

**Figure 10:**
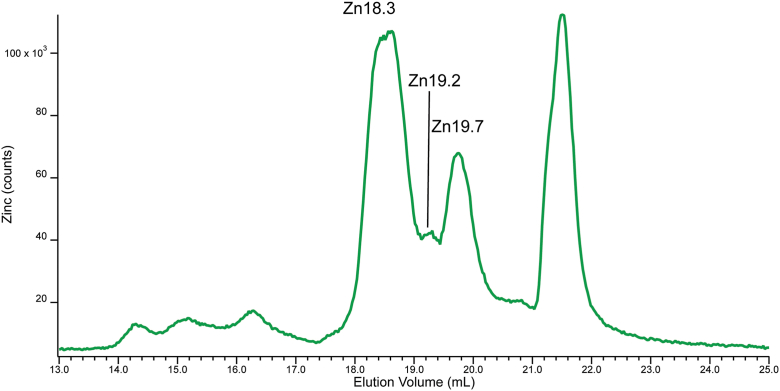
**Zn-detected chromatogram of the FTS of whole-cell lysate.** FTS, flow-through solution.

For most of this study, our focus was on the low-mass Zn pool in *cytosol* from *S. cerevisiae* cells, but we wondered whether the corresponding pool from whole-cell extracts would include additional species from other Zn pools in the cell. To probe this, cells were grown in media supplemented with 10 μM Zn(acetate)_2_. Harvested cells were lysed in buffer that excluded sorbitol (typically added to prevent non-cytosolic cellular compartments from rupturing), and fewer steps were involved prior to injection. Resulting traces (Fig. 10) exhibited all of the low-mass Zn species observed throughout the study, as well as a few additional peaks (Zn18.3, Zn19.2, Zn19.7) which might originate from non-cytosolic Zn pools such as mitochondria, vacuoles, ER *etc.* The observed relative intensities of these Zn species in the trace of Figure 10 may be more representative of their relative concentrations in within the cell.

### Effect of Zn on iron metabolism

**Figure 11:**
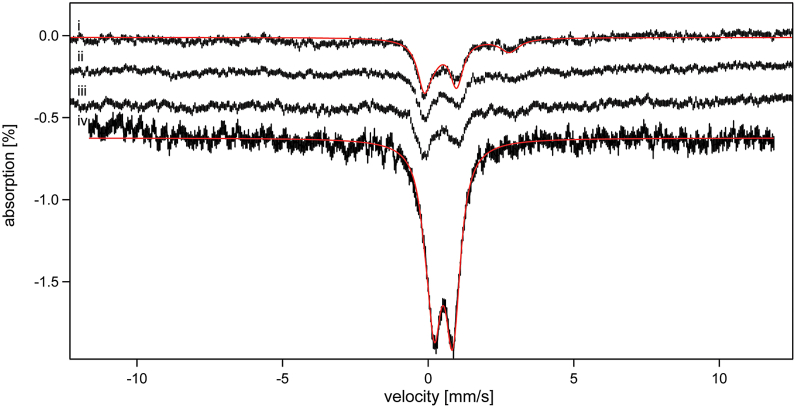
**5 K Mössbauer spectra of respiring W303 cells grown in minimal media supplemented with (*i*) 0 μM, (*ii*) 10 μM, (*iii*) 100 μM, and (*iv*) saturated Zn(acetate)_2_.** All batches contained 40 μM ^57^Fe citrate. A 0.05 T field was applied parallel to the gamma radiation. The *red line* in (*i*) is a simulation assuming two terms, one typical of [Fe_4_S_4_]^2+^ clusters (δ = 0.44 mm/s; ΔE_Q_ = 1.1 mm/s; Γ = 0.55 mm/s) at ∼ 45% of spectral intensity and one typical of nonheme high-spin Fe^II^ species (δ = 1.3 mm/s; ΔE_Q_ = 2.6 mm/s; Γ = 0.8 mm/s) at 20%. The *red line* in (*iv*) assumed that 90% of the spectral intensity arose from a doublet with δ = 0.52 mm/s; ΔE_Q_ = 0.56 mm/s; Γ = 0.5 mm/s, and that 10% arose from [Fe_4_S_4_]^2+^ clusters. Allowing the linewidth of the first term to increase to ∼ 0.8 mm/s eliminated the contribution of the second term.

Excessive nutrient Zn inhibits cell growth by inhibiting iron-sulfur cluster assembly, particularly the ISCU scaffold proteins (40, 41). To examine this effect, cells were grown under respiring conditions in minimal media containing 40 μM ^57^Fe citrate. Media was supplemented with 0 μM, 10 μM, 100 μM and saturated Zn(acetate)_2_. Resulting low-temperature low-field Mössbauer spectra (Fig. 11) were similar to previous reports (42) including a central quadrupole doublet that originates primarily from S = 0 [Fe_4_S_4_]^2+^ clusters in mitochondria and cytosol, another doublet due to high-spin Fe^II^ species in the cell, and a magnetic feature (the six-line pattern just discernible from the baseline) due to S = 5/2 Fe^III^ ions in vacuoles. Spectral signal/noise was modest because whole-cells do not contain high Fe concentrations. Nevertheless, the intensity of the central doublet clearly declined over the 0 → 10 → 100 μM series, indicating a decline of cellular iron-sulfur clusters. Cells grown in media saturated with Zn exhibited greater overall spectral intensity due to a dominant unresolved feature in the central spectral region. That dominance prevented an accurate determination of the contribution due to the central doublet, but fitting attempts suggest little if any of this species. The isomer shift and quadrupole splitting parameters required to fit the dominant feature are similar to those used to fit Fe^III^ oxyhydroxide nanoparticles as observed in mitochondria of cells that are unable to synthesize iron-sulfur clusters (42).

## Discussion

### Distribution of cellular Zn and composition of the low-mass Zn pool in *S. cerevisiae*

**Figure 12:**
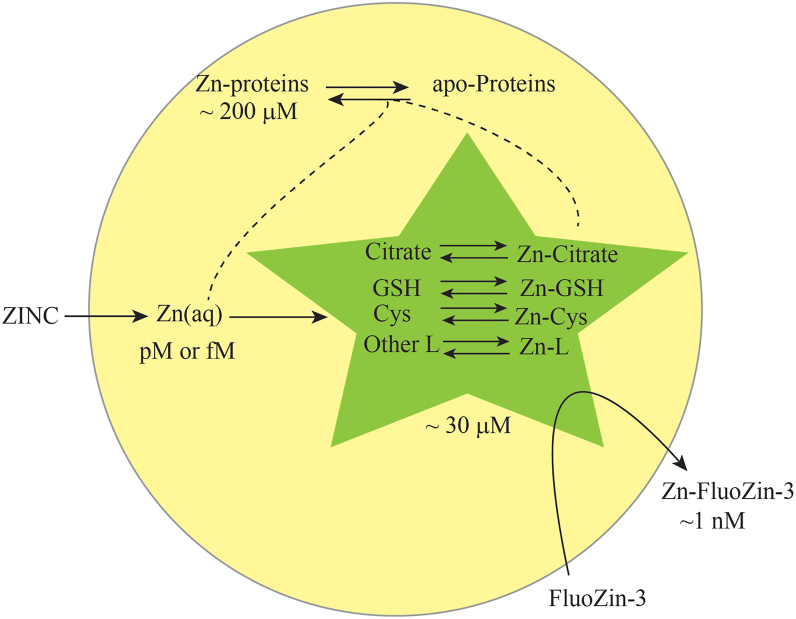
**Summary model emphasizing the low-mass Zn pool in *S. cerevisiae*.** Nutrient Zn is imported as aqueous Zn, but this rapidly is delivered to apo-proteins and nonproteinaceous low-mass ligands such that the steady-state concentration of aqueous Zn is extremely low. Low-mass ligands include citrate, glutathione, cysteine, and others that have not been identified. Approximately 85% of imported Zn binds proteins, corresponding to ∼ 200 μM, while the remainder (∼30 μM) binds low-mass ligands. Whether Zn from these low-mass complexes transfer directly to apo-proteins is uncertain. FluoZin-3 removes Zn from weakly bound complexes.

Depending on the concentration of Zn in the growth media, yeast cells distribute their intracellular Zn into Zn-bound proteins, Zn complexes in cytosol, and Zn associated with organelles such as vacuoles (Fig. 12). The vast majority of cytosolic Zn is coordinated to proteins. We propose that a tiny fraction of cellular Zn (pM or fM) may be present as *aqueous* Zn, but this would be undetectable in our experiments. We conclude that under “normal” conditions, cytosol contains a half-dozen to a dozen low-mass Zn complexes at collective concentrations of ∼ 30 μM. Under extreme conditions, the collective concentration can be as low as ∼ 2 μM and as high as ∼ 80 μM.

The observed low-mass Zn complexes appear to include Zn-citrate, Zn-cysteine, Zn-GSH, among others which could not be identified. These assignments to specific complexes are *tentative* because they are based solely on comigration with standards; we were unable to obtain confirmatory ESI-MS data despite repeated attempts.

Comparing our current results to those of *E. coli* (22) provides new insights. FTS in *E. coli* lacked the peak at 14.7 ml which we assign to Zn-citrate. In contrast, Zn-GSH was the dominating low-mass Zn species in *E. coli* (22). This could imply higher concentration of citrate and lower concentrations of GSH in *S. cerevisiae* cytosol relative to in *E. coli*, at least for the conditions used. Spiking isolated yeast FTSs with 10 μM zinc acetate increased the intensity of the Zn-citrate peak, indicating an excess of available free citrate in yeast cytosol. Interestingly, Zn-citrate dominates the low-mass Zn species in breast milk, coconut water, and other plant extracts (43, 44, 45). Differences in binding strengths between Zn-citrate and Zn-GSH along with differences in cellular concentrations of the free ligands likely explain these differences. As mentioned above, GSH may decompose faster in yeast than in *E. coli* which may contribute to the observed differences. The peak assigned to a Zn-cysteine complex was evident in nearly all chromatograms of both *S. cerevisiae* and *E. coli*, suggesting that this complex is a consistent (but not dominating) component of the low-mass Zn pools in both organisms.

The collective size of low-mass Zn complexes in yeast was in the μM range, similar to that observed for *E. coli* cytoplasm. These concentrations are orders-of-magnitude higher than previous reports of pM or fM for labile Zn pool concentrations. Our spiking experiments show that the yeast cytosol can accommodate *at least* 15 μM aqueous Zn ions, similar to the situation in *E coli*. This significant capacity explains the near absence of *aqueous* (sometimes incorrectly called “free”) Zn in both cells. It also indicates an unexpectedly large Zn *buffer depth*, as introduced by Krezel and Maret (21). As shown previously (22), the size of the low-mass Zn pool is not controlled by the Irving-Williams series, in contrast with the situation for aqueous Zn. Rather, the size of the low-mass pool should be controlled by the binding/dissociation constants for Zn complexes (*e.g.* Zn-citrate, Zn-GSH, and Zn-cysteine) and the concentration of available ligands in the cell.

### Unexpected targets of Zn chelator probes

FluoZin-3 is a popular fluorescence-based chelator probe that has been used to evaluate the labile Zn pool in a variety of cells. Our results with FluoZin-3 and those obtained previously with TPEN (22) illustrate the serious problems in using these probes to investigate labile metal pools. These probes are commonly assumed to react mainly with low-mass Zn complexes (17, 18, 20, 21), but their reactivity is far more diverse. TPEN mainly reacts with Zn bound to proteins, not low-mass Zn complexes (22), and FluoZin-3 is unable to remove Zn from either proteins or strong Zn coordination complexes (this study). Thus, the size estimates of such pools will vary widely according to the strength of the probe used, which renders such estimates unreliable and of questionable utility.

Here we incubated isolated cytosol with as high as 100 μM FluoZin-3, and found that it bound only ∼ 1.5% of the ∼ 230 μM of Zn in yeast cytosol. However, in other studies whole cells are typically incubated with only about 1/50th of that concentration of FluoZin-3 (46, 47). Assuming proportionally less binding suggests that only ∼ 70 nM (230 μM × 0.015 ÷ 50) would be coordinated under those conditions. Moreover, of the FluoZin-3 *added* to cells, only a fraction likely *penetrates* cells, in which case only a few nM or hundreds of pM Zn might have actually been coordinated. This is a gross underestimate of the true size of the low-mass Zn pool but it is within an order-of-magnitude of the reported concentrations of “free” Zn in cells using such probes.

### Homeostatic regulation of the low-mass Zn pool in *S. cerevisiae*

Our results reveal that yeast cells regulate their cytosolic Zn concentrations more effectively than do *E. coli,* perhaps due to their ability to sequester Zn in vacuoles and bind/unbind to proteins in the cell. Both vacuoles and proteins would seem to be used as buffers. The total zinc concentrations of the cell did increase in yeast with increasing zinc supplementation, similar to the situation in *E. coli*. However, the whole cell Zn concentrations for yeast were much lower than in *E coli*, possibly due to less zinc nonspecifically binding to the outer wall of yeast than to the *E. coli* exterior.

We attempted to perturb the low-mass Zn pool in yeast in various ways. We grew cells on minimal media supplemented with 0, 10, and 100 μM Zn(acetate)_2_ but this had only modest effect, far less than observed with *E coli*. We spiked CYT and FTS samples with ^67^Zn and found a strong buffering capacity of these solutions, such that Zn peak intensities only increased moderately. We compared mutant cells (lacking the vacuolar importers Zrc1 and Cot1), expecting major increased intensities of cytosolic Zn, but this was not observed. We grew WT cells under extreme Zn conditions, but the effect on the low-mass Zn pool was modest. The collective lesson is that *S. cerevisiae* cells regulate Zn with remarkable effectiveness.

The flip-side of this issue is that the threshold for Zn toxicity might not be much higher than the upper limit of what we observed. For example, our mutant cells were quite sensitive to Zn in the growth media (75 μM Zn in fermenting and 50 μM Zn in respiring), such that concentrations of nutrient Zn higher than these levels were lethal in the *zrc1cot1ΔΔ* strain. Thus, we were unable to investigate the molecular origins of the toxicity. Despite growing these cells in zinc supplementations as close to the toxic threshold as possible (50 μM for fermenting), the cells showed only modest changes in the cytosolic zinc concentrations and minor changes in the LC-ICP-MS intensities of the species. Cells can tolerate higher (μM) concentrations than previous estimates essentially because the Zn coordination complexes that compose the pool bind Zn far more tightly (and with lower kinetic lability) than aqueous Zn ions, which limits toxicity.

### Effect of excess Zn on iron metabolism in *S. cerevisiae*

Our Mössbauer study allowed the effect of excess Zn on iron metabolism to be explored. Our results confirmed earlier studies that excess Zn damages iron-sulfur clusters in proteins or inhibits their assembly. The observation of a Mössbauer spectral feature with properties typical of Fe^III^ oxyhydroxide nanoparticles (42) prompts speculation that the inhibition of iron-sulfur-cluster assembly by excess Zn might activate the Aft1/2-dependent iron regulon, leading to the formation of nanoparticles. Further studies are required to test this intriguing hypothesis.

## Experimental procedures

### Cell growths

Thirty-two batches of wild-type W303 cells were cultured aerobically in one or 2 L of fermenting minimal media supplemented with 1 mM EDTA or 0, 10, 100, or 6000 μM Zn(acetate)_2_. Fifty milliliter precultures were inoculated with cells and allowed to grow for 24 h (fermenting) at 30 °C with 180 rpm shaking. The cultures were then transferred to 1 or 2 L of minimal media and allowed to grow to a final OD_600_ of 0.6 to 1.2. Cells were spun at 5000*g* for 5 min and then resuspended using double-distilled water. The suspension was spun at 5000*g* for 5 min to repellet the cells. This wash procedure was repeated. After the final wash solution was discarded, the mass of the pellet was recorded. Three batches each of CM100 (*MAT*α *can1-100 his3-11,15 leu2-3112 trp1-1 ura3-52*) and CM104 (*MAT*α *can1-100 his3-11,15 leu2-3112 trp1-1 ura3-52 zrc1::HIS3 cot1::URA3*) cells were cultured in minimal media to the same OD_600_ of 0.6 to 1.2 with 50 μM Zn(acetate)_2_.

### Mössbauer spectroscopy

Four sets of respiring media were prepared containing 0, 10, 100, and 6000 μM Zn(acetate)_2_, respectively. Cells were supplemented with 40 μM ^57^Fe(III) citrate (^57^Fe from Isoflex), grown as above, and pelleted into Mössbauer cups at 12,281*g* for 10 min after washing twice with double distilled water. The instrument has been described (42). Spectra were calibrated to α-iron foil at RT and fitted using WMOSS software.

### Cytosol Isolations

This basic procedure has been described (48). Cells were resuspended in 10 mM DTT 20 mM ammonium bicarbonate (ABC) pH 7.5 in distilled water (5 ml/g of pellet) and incubated for 15 min at 30 °C with 180 rpm shaking. The sample was centrifuged at 4000*g* for 5 min and the supernatant was poured off in the glovebox. The supernatant was discarded and the pellet was resuspended in ABC buffer (20 mM ammonium bicarbonate, 0.6 M sorbitol pH 7.5 in HPW) to the extent of 5 ml per 1 g of cell pellet. Zymolyase (1.5 mg zymolyase/g of cell pellet) was also added. The suspension was incubated for 30 min at 30 °C with 180 rpm shaking. Aliquots of the suspension pre- and post-zymolyase addition were used to monitor zymolyase activity. The OD_600_ of the two aliquots were measured until the post-zymolyase aliquot was ∼30% of the pre-zymolyase sample. The suspension was then centrifuged at 1800*g* for 5 min. The sample was resuspended in ABC buffer (5 ml/1 g of cell pellet) along with a final concentration of 10 mM PMSF (Thermo Fisher Scientific). Approximately 15 ml of the cell suspension was transferred to a 40 ml capacity glass Dounce homogenizer (Kimble) and was homogenized for 10 strokes with pestle A. This process was repeated as needed for the entire cell suspension to generate whole cell lysate. A 1 ml aliquot of the whole cell lysate was stored at −80 °C for metal analyses. The sample was removed from the glovebox and centrifuged at 1000*g* for 15 min. The supernatant was transferred to a fresh tube and then centrifuged at 15,000*g* for 30 min. The sample was brought into the glovebox and the supernatant was transferred to ultracentrifuge tubes. The samples were centrifuged for 1 h at 112,000*g*. The supernatant was transferred to fresh centrifuge tubes and centrifuged at 185,000*g* for 2.5 h. The samples were filtered using either an Ultracel Amicon Ultra 2 ml regenerated cellulose 3 kDa centricon filter to generate FTS samples or a Titan 0.2 μm syringe filter to generate CYT samples. Samples were analyzed by Western blot for vacuolar and mitochondrial contamination (Fig. S1).

### Metal analyses

A set of 5 ICP-MS standards were prepared *via* 10-fold serial dilution of TEXSAM-15REV3 (Inorganic Ventures). The standards had a final concentration of 5% trace-metal-grade HNO_3_ (Thermo Fisher Scientific). Two blanks of HPW were prepared with 5% HNO_3_. For elemental analysis of the samples, three aliquots of the resulting CYT, FTS, or whole cell lysates were transferred to 15 ml polypropylene tubes (Agilent). One hundred and fifty microliters of trace-metal-grade HNO_3_ was added to each tube. Tubes were capped, sealed with electrical tape, and incubated at 80 °C for 24 h. Analyses were performed on an Agilent 8900 ICP-MS with reaction cell gases set to 2.0 ml/min H_2_ 30% O_2_. An internal standard IV-ICPMS-71D (Inorganic Ventures) was prepared in 5% HNO_3_. Back-calculations to concentrations in whole cells were performed as described (48).

### LC-ICP-MS

A standard metal stock solution of 1 mM Zn(acetate)_2_ dihydrate (Acros Organics) was prepared in HPW. Ten millimolar ligand stock solutions of sodium citrate (Thermo Fisher Scientific), reduced GSH (Sigma Aldrich), and L-cysteine (Sigma Aldrich) were prepared in HPW. Zn-ligand standards were diluted to final concentrations of 2 μM Zn and 1 mM of each ligand in 20 mM ABC (Sigma Aldrich) pH 7.5. To match the concentrations of ^23^Na, ^24^ Mg, and ^39^K in the isolated FTS samples, metal ligand standards were spiked with 100 mM stocks of NaCl (Sigma Aldrich), MgCl_2_ hexahydrate (Thermo Fisher Scientific), and KCl (EMD) to obtain final concentrations of 0.5 mM, 0.2 mM, and 0.9 mM, respectively.

One hundred microliters of standards, CYT, or FTSs were injected onto a Superdex 30 Increase column (Cytiva) installed in an Agilent 1260 LC system with a bio-inert quaternary pump (G5611A), multisampler (G5688A), diode array (G4212B), and fraction collector (G5664A), all housed within a refrigerated glovebox (MBraun Labmaster). The LC was connected in tandem to the ICP-MS (external to the box) to collect elemental data for ^32^S, ^64^Zn, ^66^Zn, ^67^Zn, and ^68^Zn. The counts for the zinc isotopes were summed to obtain total zinc counts. The mobile phase was 20 mM ammonium bicarbonate (ABC) pH 7.5 that had been filtered with a 0.22 μm polyethersulfone stericup filter (Corning) and then degassed on a Schlenk line. Flow rate was 0.6 ml/min and data were collected for 1 h per sample. Between sample injections, the column was cleaned by injecting 100 μl of a chelator cocktail consisting of 500 μM each of EDTA (Sigma Aldrich), ethylene glycol-bis(β-aminoethyl EGTA (Sigma-Aldrich), 1,10-phenanthroline (phen) (Acros Organics), 2,2-bipyridine (Alfa Aesar), bathocuproinedisulfonic acid (Sigma Aldrich), deferoxamine (EMD Millipore), TPEN (Sigma-Aldrich), and 10 mM ascorbic acid (Acros Organics). This was followed by injecting 100 μl of HPW to wash the column of chelator cocktail. This washing procedure was repeated between sample injections. Analyses were performed with reaction cell gases as described above.

### Zn spiking

A stock solution of 100 μM ^67^ZnSO_4_ (Isoflex) was prepared in HPW. One hundred and fifty μL of CYT or FTS samples were spiked with this zinc solution to obtain a final concentration of 2, 5, 10, 20, or 50 μM added ^67^Zn and this solution was allowed to react in the glovebox for at least 3 h. Counts for ^67^Zn and ^32^S were adjusted to account for dilution.

### FluoZin-3 spiking

A stock solution of 1 mM Fluozin-3 cell permeant (Sigma Aldrich) was prepared in DMSO. One hundred 50 μL of CYT samples were spiked with this Fluozin-3 solution to obtain a final concentration of 13.3, 20, 50, or 100 μM Fluozin-3 and allowed to react in the glovebox for at least 3 h. Counts for total zinc and absorbance at 494 nm were back-calculated to account for dilution.

## Data availability

All data are contained within the manuscript and SI.

## Supporting information

This article contains supporting information. The file called SI Data and Fits includes: Figure 2 data; Figure 3 data; Figures 2 and 3 summary; Figure 4 data; Figure 4 summary; Figure 5 data; Figure 5 summary; Figure 6 data; Figure 6 summary; Figure 7 data; Figure 7 summary; Figure 8 data; Figure 8 summary; Figure 9 data; Figure 9 summary; Figure 10 data; Figure 10 summary. The file called Supplemental Information includes Figure S1, Western blot of three isolated cytosol batches.

## Conflict of interest

The authors declare that they have no conflicts of interest with the contents of this article.

## Supporting information

Data and Fits

Figure S1

**Abbreviations**
ABC, ammonium bicarbonate
FTS, flow-through solution
GSH, glutathione
LMM, low-molecular-mass
TPEN, N,N,N′,N′tetrakis(2-pyridinylmethyl)-1,2-ethanediamine

## References

[1] Eide D.J. (2009). Homeostatic and adaptive responses to zinc deficiency in *Saccharomyces cerevisiae*. J. Biol. Chem..

[2] Eide D.J. (2020). Transcription factors and transporters in zinc homeostasis: lessons learned from fungi. Crit. Rev. Biochem. Mol. Biol..

[3] Maret W. (2025). The arcana of zinc. J. Nutr..

[4] Maret W. (2024). Chemistry meets biology in the coordination dynamics of metalloproteins. J. Inorg. Biochem..

[5] Bird A.J., Wilson S. (2020). Zinc homeostasis in the secretory pathway in yeast. Curr. Opin. Chem. Biol..

[6] Fukada T., Kambe T. (2018). Welcome to the world of zinc signaling. Int. J. Mol. Sci..

[7] Wang J., Zhao H., Xu Z., Cheng X. (2020). Zinc dysregulation in cancers and its potential as a therapeutic target. Cancer Biol. Med..

[8] Szewczyk B. (2013). Zinc homeostasis and neurodegenerative disorders. Front. Aging Neurosci..

[9] Plum L.M., Rink L., Haase H. (2010). The essential toxin: impact of zinc on human health. Int. J. Environ. Res. Public Health.

[10] Wang Y., Weisenhorn E., MacDiarmid C.W., Andreini C., Bucci M., Taggart J. (2018). The cellular economy of the *Saccharomyces cerevisiae* zinc proteome. Metallomics.

[11] Cyert M.S., Philpott C.C. (2013). Regulation of cation balance in *Saccharomyces cerevisiae*. Genetics.

[12] Andreini C., Banci L., Bertini I., Rosato A. (2006). Zinc through the three domains of life. J. Proteome Res..

[13] MacDiarmid C.W., Gaither L.A., Eide D.J. (2000). Zinc transporters that regulate vacuolar zinc storage in *Saccharomyces cerevisiae*. EMBO J..

[14] Jorgensen P., Nishikawa J.L., Breitkreutz B.J., Tyers M. (2002). Systematic identification of pathways that couple cell growth and division in yeast. Science.

[15] Pick A.M., Weber K., Jacobs M.F., Carlson J., Wittman S., Fahrer J. (2025). A bright spiropyran-based zinc sensor for live-cell imaging. ACSOmega.

[16] Aron A.T., Ramos-Torres K.M., Cotruvo J.A., Chang C.J. (2015). Recognition- and reactivity-based fluorescent probes for studying transition metal signaling in living systems. Acc. Chem. Res..

[17] Wang F., Wang K., Kong Q., Wang J., Xi D., Gu B. (2021). Recent studies focusing on the development of fluorescence probes for zinc ion. Coord. Chem. Rev..

[18] Pratt E.P.S., Damon L.J., Anson K.J., Palmer A.E. (2021). Tools and techniques for illuminating the cell biology of zinc. Biochim. Biophys. Acta - Mol. Cell Res..

[19] Vinkenborg J.L., Nicolson T.J., Bellomo E.A., Koay M.S., Rutter G.A., Merkx M. (2009). Genetically encoded FRET sensors to monitor intracellular Zn^2+^ homeostasis. Nat. Methods.

[20] Outten C.E., O'Halloran T.V. (2001). Femtomolar sensitivity of metalloregulatory proteins controlling zinc homeostasis. Science.

[21] Krezel A., Maret W. (2006). Zinc-buffering capacity of a eukaryotic cell at physiological pZn. J. Biol. Inorg. Chem..

[22] Kreinbrink A.C., Romano N., Hierholzer J.D., Lindahl P.A. (2025). Low-mass zinc pools in *Escherichia coli*: micromolar concentrations, diverse compositions, and Zn-glutathione dominating under Zn-replete conditions. J. Biol. Chem..

[23] Krezel A., Maret W. (2016). The biological inorganic chemistry of zinc ions. Arch. Biochem. Biophys..

[24] Nguyen T.Q., Kim J.E., Brawley H.N., Lindahl P.A. (2020). Chromatographic detection of low-molecular-mass metal complexes in the cytosol of *Saccharomyces cerevisiae*. Metallomics.

[25] Zhao Y.Y., Cao L.C., Liu L.Y., Wang J., Li J., Li S.Y. (2020). Identification of the genetic requirements for zinc tolerance and toxicity in *Saccharomyces cerevisiae*. G3.

[26] Wilson S., Bird A.J. (2016). Zinc sensing and regulation in yeast model systems. Arch. Biochem. Biophys..

[27] Sun A., Wang W.X. (2022). Insights into the kinetic regulation of Zn bioaccumulation at trace levels: lighting up *Saccharomyces cerevisiae*. Chemosphere.

[28] Lyons T.J., Gasch A.P., Gaither L.A., Botstein D., Brown P.O., Eide D.J. (2000). Genome-wide characterization of the Zap1p zinc-responsive regulon in yeast. Proc. Natl. Acad. Sci. U. S. A..

[29] Zhao H., Eide D. (1996). The yeast ZRT1 gene encodes the zinc transporter protein of a high-affinity uptake system induced by zinc limitation. Proc. Natl. Acad. Sci. U. S. A..

[30] Waters B.M., Eide D.J. (2002). Combinatorial control of yeast FET4 gene expression by iron, zinc, and oxygen. J. Biol. Chem..

[31] Jensen L.T., Ajua-Alemanji M., Culotta V.C. (2003). The *Saccharomyces cerevisiae* high affinity phosphate transporter encoded by PHO84 also functions in manganese homeostasis. J. Biol. Chem..

[32] Rosenfeld L., Reddi A.R., Leung E., Aranda K., Jensen L.T., Culotta V.C. (2010). The effect of phosphate accumulation on metal ion homeostasis in *Saccharomyces cerevisiae*. J. Biol. Inorg. Chem..

[33] Pagani A., Villarreal L., Capdevila M., Atrian S. (2007). The *Saccharomyces Cerevisiae* Crs5 metallothionein metal-binding abilities and its role in the response to zinc overload. Mol. Microbiol..

[34] Simm C., Lahner B., Salt D., LeFurgey A., Ingram P., Yandell B. (2007). *Saccharomyces Cerevisiae* vacuole in zinc storage and intracellular zinc distribution. Eukaryot. Cell.

[35] MacDiarmid C.W., Milanick M.A., Eide D.J. (2003). Induction of the ZRC1 metal tolerance gene in zinc-limited yeast confers resistance to zinc shock. J. Biol. Chem..

[36] Liuzzi J.P., Guo L., Yoo C.W., Stewart T.S. (2014). Zinc and autophagy. Biometals.

[37] Falchuk K.H. (1998). The molecular basis for the role of zinc in developmental biology. Mol. Cell. Biochem..

[38] Baudouin-Cornu P., Lagniel G., Kumar G., Huang M.E., Labarre J. (2012). Glutathione degradation is a key determinant of glutathione homeostasis. J. Biol. Chem..

[39] Gitan R.S., Luo H., Rodgers J., Borderius M., Eide D. (1998). Zinc-induced inactivation of the yeast ZRT1 zinc transporter occurs through endocytosis and vacuolar degradation. J. Biol. Chem..

[40] Li J., Ren X., Fan B., Huang Z., Wang W., Zhou H. (2019). Zinc toxicity and iron-sulfur cluster biogenesis in *Escherichia coli*. Appl. Environ. Microbiol..

[41] Fox N.G., Martelli A., Nabhan J.F., Janz J., Borkowska O., Bulawa C. (2018). Zinc(II) binding on human wild-type ISCU and Met140 variants modulates NFS1 desulfurase activity. Biochimie.

[42] Fernandez S., Wofford J.D., Shepherd R.E., Vali S.W., Dancis A., Lindahl P.A. (2022). Yeast cells depleted of the frataxin homolog Yfh1 redistribute cellular iron: studies using Mössbauer spectroscopy and mathematical modelling. J. Biol. Chem..

[43] Alchoubassi G., Kińska K., Bierla K., Lobinski R., Szpunar J. (2021). Speciation of essential nutrient trace elements in coconut water. Food Chem..

[44] Sowik I., Zajda J., Ruzik L. (2023). Speciation analysis of copper and zinc in plant-based drinks using hyphenated techniques. Microchem. J..

[45] Milačič R., Ajlec D., Zuliani T., Žigon D., Ščančar J. (2012). Determination of Zn-Citrate in human milk by CIM monolithic chromatography with atomic and mass spectrometry detection. Talanta.

[46] Han Y., Goldberg J.M., Lippard S.J., Palmer A.E. (2018). Superiority of SpiroZin2 versus FluoZin-3 for monitoring vesicular Zn^2+^ allows tracking of lysosomal Zn^2+^ pools. Sci. Rep..

[47] Ali S., Cuajungco M.A. (2020). Protocol for quantifying zinc flux in cultured cells using fluorescent indicators. Star Protoc..

[48] Kim J.E., Jeon S.Y., Lindahl P.A. (2023). Discovery of an unusual copper homeostatic mechanism in yeast cells respiring on minimal medium and an unexpectedly diverse labile copper pool. J. Biol. Chem..

