# Supplementary material for: Approximate size and preliminary composition of the low-mass zinc pool in the cytosol of Saccharomyces cerevisiae
Source: J Biol Chem. 2026 Mar 20;302(5):111394. doi: 10.1016/j.jbc.2026.111394 (PMC13092671; doi:10.1016/j.jbc.2026.111394)
Supplement: Figure S1 [file mmc2.docx]

**Supplemental Information**

Title: Approximate size and preliminary composition of the low-mass zinc pool in the cytosol of *Saccharomyces cerevisiae*

Authors: Alexia C. Kreinbrink and Paul A. Lindahl

Figure S1: Western Blot of three isolated cytosol batches


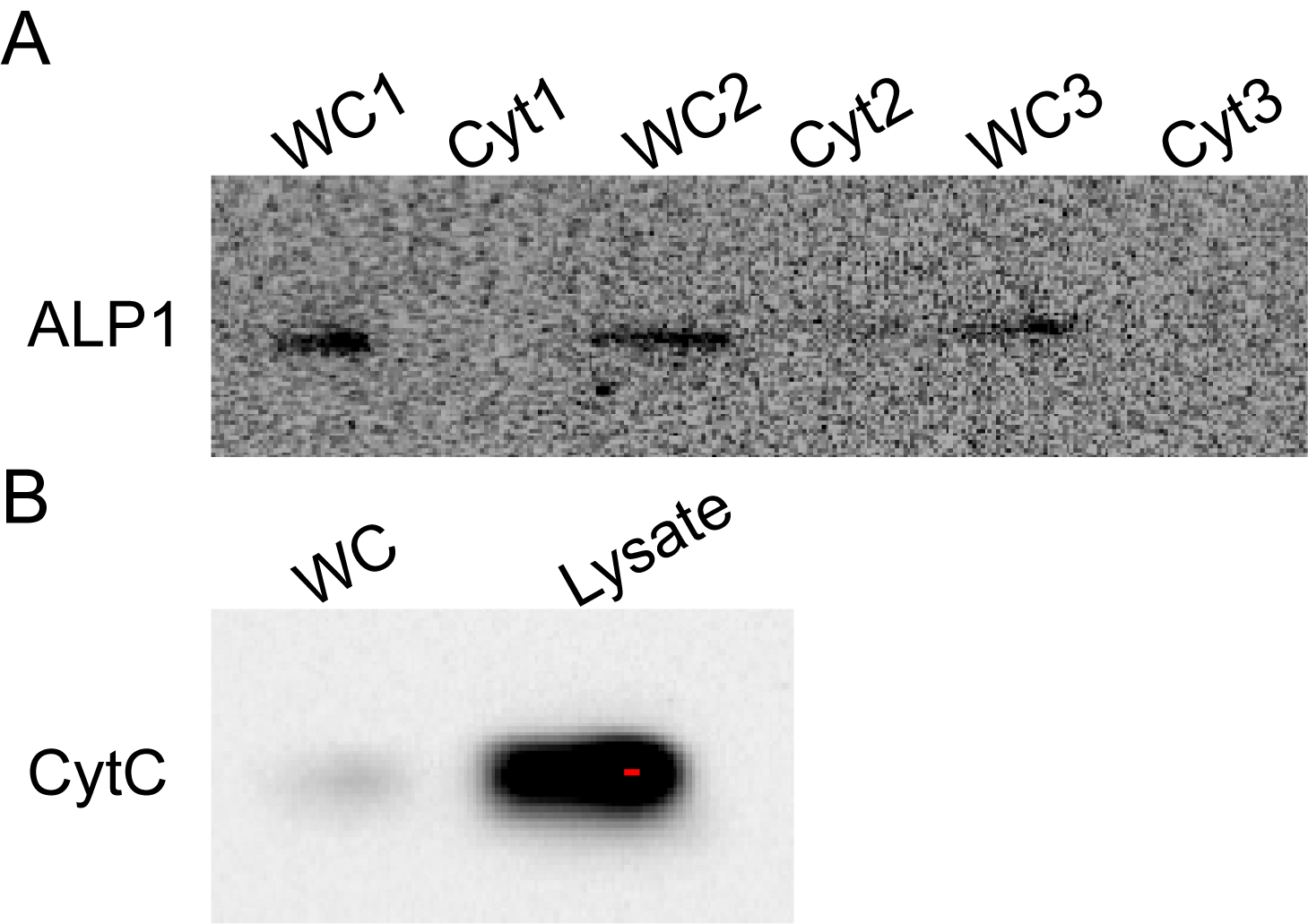


Figure S1: Western blot analysis of whole cells, cell extracts, and isolated cytosol. Protein concentrations were quantified by Pierce™ BCA Protein Assay Kit (Thermo Scientific™). Samples were run on a NuPAGE™ 10% Bis–Tris protein gel (Invitrogen™). Separated proteins were transferred to PVDF membranes using a Trans-Blot transfer cell (Bio-Rad). Membranes were blocked with 5% milk dissolved in Tris-buffered saline with 0.1% Tween (TBST-milk) for 1 h at RT before incubating with primary antibodies overnight at 4 °C. All primary antibodies were prepared in TBST-milk at the following dilutions: 1 : 500 of anti-ALP antibody for vacuole marker (Abcam, 1D3A10) and 1 : 1000 of anti-CytC antibody for mitochondrial marker (Life Technologies, 37BA11). Goat anti-mouse IgG HRP-conjugated secondary antibody was from Invitrogen (clone G-21040). Both secondary antibodies were used at 1 : 5000 dilution for 1 h at RT. Clarity Max™ Western ECL (Panel A) or Clarity™ Western ECL Substrate (Panel B) (Bio-Rad) was added, and 10 images were obtained with standard acquisition mode from 10-600 seconds.

Panel A: 82.5 μg of whole cells and isolated cytosol from 6 mM Zn supplemented cells. None of the cytosol samples indicated vacuolar contamination. Cytochrome c bands were not observed likely due to having grown cells under fermentation conditions.

Panel B, 35 μg of whole respiring cells and concentrated cell lysate from 0 μM Zn respiring conditions, using the cytochrome c antibody as a positive control.
